# Analysis of Structural Heterogeneity in Low-Rank Coal and Its Pyrolyzed Char Using Multi-Point Scanning Micro-Raman Spectroscopy

**DOI:** 10.3390/molecules29102361

**Published:** 2024-05-17

**Authors:** Yaqi Gao, Chong Zou, Yuan She, Zhengyan Huang, Siqi Li

**Affiliations:** College of Metallurgical Engineering, Xi’an University of Architecture and Technology, Xi’an 710311, China; gyq99@xauat.edu.cn (Y.G.); sheyuan@xauat.edu.cn (Y.S.); huangzhengyan@xauat.edu.cn (Z.H.); lisiqi990321@163.com (S.L.)

**Keywords:** micro-Raman spectroscopy, low-rank coal, pyrolysis, char, mapping

## Abstract

Understanding the changes in carbon structure during the mid–low-temperature pyrolysis of low-rank coal is important for efficient utilization. Raman spectroscopy is commonly used to analyze the structural order of carbonaceous materials, but traditional methods may overlook the heterogeneity of coal/char. This research explores the heterogeneity of char structure derived from low-rank coal at 700 °C through multi-point micro-Raman analysis. The analysis of parameters such as area (*A*), intensity (*I*), full width at half maximum (*FWHM/W*), and peak position (*P*) reveals that the carbon structure becomes less ordered as coal transforms into char due to the deposition of small molecules on the surface. The study emphasizes the benefits of multi-point detection for gaining in-depth insights into the structural evolution of carbonaceous materials. The increased standard deviation of Raman parameters indicates diverse structural characteristics resulting from pyrolysis at this temperature, which traditional methods may not capture effectively. The mapping method used in this research visually illustrates the distribution of carbon structures in the region.

## 1. Introduction

Raman spectroscopy is a method used to analyze the scattering spectra of materials under different frequencies of incident light through the Raman scattering effect. This technique provides valuable information about molecular vibration and rotation, making it useful for molecular structure research [1,2]. Due to its ability to identify the degree of order in carbonaceous materials, Raman spectroscopy has found extensive applications in the characterization of carbonaceous material [3,4,5,6,7]. Raman structural parameters can be combined with maturity assessments to reflect the structural characteristics of coal [8,9]. The utilization of coal often involves pyrolysis, which directly impacts the microstructural re-ordering of char. Therefore, it is crucial to understand the mechanism of coal pyrolysis and optimize the chemical structure of the resulting product by adjusting the temperature. Many scholars have dedicated their research to this particular area [10,11]. In recent decades, scholars have identified features such as aromatic rings and various carbon-containing functional groups present in the process of pyrolysis of coal [12,13,14,15,16,17]. First-order Raman spectroscopy and second-order Raman spectroscopy were used to comprehensively analyze the structural changes in coal and char [18,19,20,21,22,23,24,25,26,27,28,29]. However, given the heterogeneous nature of coal/char, there is a need for advanced technologies to provide further characterization. It has been proposed that a statistically sufficient number (n > 30) of spectra should be collected to better explain the frequency distribution of the detected structural types [30]. As a result, some scholars have started to explore new methods for the investigation of coal/char [31].

Laser confocal micro-Raman spectroscopy offers the benefits of conventional Raman spectroscopy along with excellent spatial resolution. By focusing incident light on a micro-region of the sample using a microscopic objective lens, this technique enables the accurate acquisition of Raman spectral information related to the chemical composition, crystal structure, defects, and crystallinity of a specific area, without any interference from the surrounding components [32,33,34,35,36,37]. This obtains more sample points. Parameters of a large number of points can be used to construct microscopic distribution images of physical and chemical properties such as composition and defects of the sample. Micro-Raman imaging is considered advantageous compared to other detection methods as it provides a wealth of valuable information [38,39,40,41,42]. Previous studies have utilized micro-Raman to analyze the structure of coal and have successfully achieved the two-dimensional planar chemical imaging of coal as well as in situ identification of coal macerals based on the intensity parameters of D, G, and V peaks [43]. However, micro-Raman spectroscopy has not been well used to characterize char structural changes during coal pyrolysis. Therefore, it is of great significance to further establish a method for analyzing coal/coke structure by micro-Raman spectroscopy, whether it is from the need to develop methods for characterizing heterogeneous carbonaceous materials or from the in-depth understanding of the pyrolysis mechanism of coal.

In this study, low-rank coal and its prepared char were used as raw materials and analyzed using micro-Raman spectroscopy. This paper highlights the benefits of utilizing micro-Raman spectroscopy for multi-point scanning, rather than delving into the specific structural evolution from coal to coke. Therefore, only char at a single temperature was chosen for analysis. Additionally, given the varied Raman parameters employed by different researchers, multiple angle parameters were selected to facilitate multidimensional exploration. Hence, the area parameter (*A*), intensity parameter (*I*), half-height width parameter (*FWMH/W*), peak position parameter (*P*), and correlation between these parameters were used to create a map. These maps provided a visual representation of the changes in carbon structure during the conversion of low-rank coal to char. Additionally, the study compared the differences in results obtained from different sampling points.

## 2. Results and Discussion

**Figure 1:**
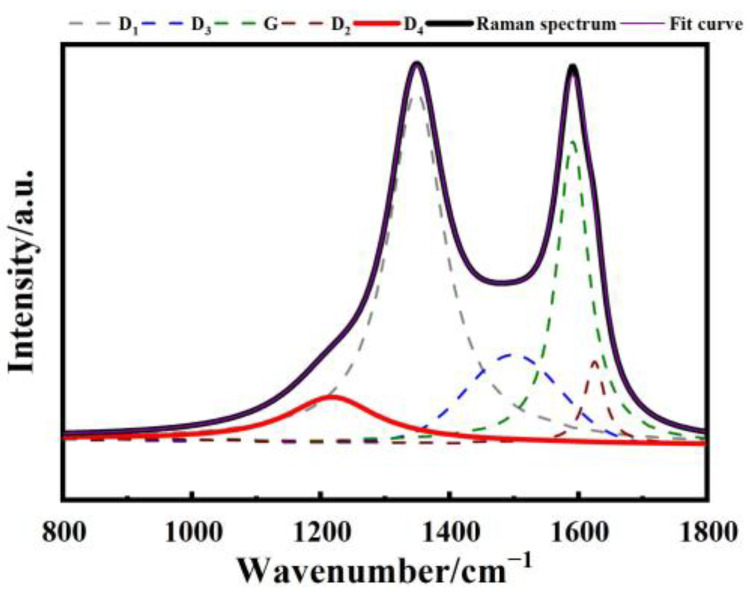
Curve fitting for the Raman spectra.

The first-order Raman spectrum of coal (800–1800 cm^−1^) exhibits two characteristic peaks: the G peak at 1580 cm^−1^ and the D (D_1_) peak at 1350 cm^−1^ [44]. The G peak corresponds to stretching vibration of the C=C bond in the graphite lattice plane [45], indicating a strong chemical bond formed by the sp^2^ hybridization of carbon atoms. It represents the E_2g2_ vibration mode of perfect graphite, resulting from the breathing vibration of the aromatic ring [46]. On the other hand, the D peak is attributed to defects in the graphite lattice, such as edge disordered arrangement and low symmetrical carbon structure. It is considered a defect peak. Sadezky et al. [47] conducted a study on the Raman spectra of carbonaceous materials with low structural order and proposed a five-peak fitting method for the first-order peak region. Sadezky tested nine different combinations of Lorentz and Gaussian fitting during the fitting process, ultimately identifying the optimal fitting scheme, as detailed in Table 1. This method is widely utilized for the Raman analysis of coal. Along with the G and D peaks, there are additional peaks labeled as D_4_, D_3_, and the D_2_ at 1200 cm^−1^, 1500 cm^−1^, and 1620 cm^−1^, respectively. These peaks have distinct vibration modes, as summarized in Table 1. Figure 1 illustrates the effectiveness of the five-peak fitting.

The D_2_ peak is believed to be generated by sp^2^-mode amorphous carbon, such as from organic molecules, fragments, or functional groups in highly disordered carbonaceous materials [48]. The D_3_ peak is more commonly found in low-rank coal with a high degree of disorder, and it may be caused by the stretching vibration of C–C in an aliphatic structure or an olefin-like structure [49]. However, there is still controversy surrounding its attribution [50]. The D_4_ peak is formed due to the mixed sp^2^–sp^3^ hybrid structure at the edge of the crystallite or the presence of C=C and C–C bonds, which represent reactive sites [51,52]. In this study, the data after the peak fitting of the Raman spectrum were calculated, and the ratio of the area (*A_D2+D3+D4_/A_all_*, *A_D1_/A_G_*, *A_D2_/A_all_*, *A_D3_/A_all_*, *A_D4_/A_all_*), the ratio of intensity (*I_D1_/I_G_*, *I_D2_/I_G_*, *I_D3_/I_G_*, *I_D4_/I_G_*), the FWMH of the G peak (*W_G_*), and the position of peaks (*P_G-D_*) were obtained.

### 2.1. Area Parameter (A)

**Figure 2:**
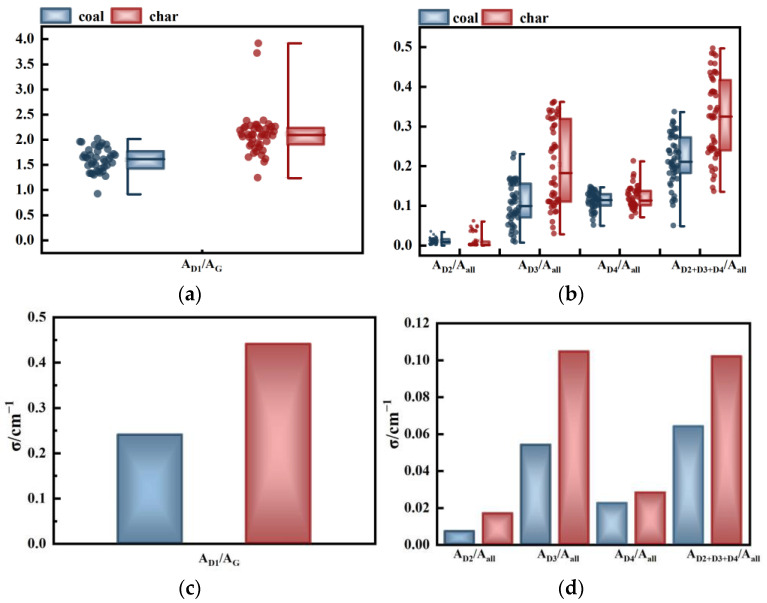
Relationship between the area parameters of coal and char: (**a**) change in *A_D1_/A_G_*; (**b**) changes in *A_D2_/A_all_*, *A_D3_/A_all_*, *A_D4_/A_all_*, and *A_D2+D3+D4_/A_all_*; (**c**) standard deviation of *A_D1_/A_G_*; and (**d**) standard deviations of *A_D2_/A_all_*, *A_D3_/A_all_*, *A_D4_/A_all_*, and *A_D2+D3+D4_/A_all_*.

Figure 2 illustrates the variation relationship and standard deviation of coal and coke area parameters. In Figure 2a, the change in *A_D1_/A_G_* parameter between coal and char is presented, and the mean value is indicated by the center line of the half box chart box. It can be observed that the value of char is greater than that of coal. The area of the G peak signifies the content of the perfect graphite structure, while the D_1_ band is associated with the vibration mode of the graphite lattice A_1g_, indicating the presence of in-plane defects and heteroatoms. Hence, the higher mean value of *A_D1_/A_G_* of char in Figure 2a suggests that the content of disordered structure in coal is higher than that of perfect graphite during medium and low temperature pyrolysis. Additionally, it can be seen from Figure 2b that the mean value of *A_D3_/A_all_* is also increasing, and the increase is more significant. The appearance of the D_3_ band can be attributed to the gap defects located outside the aromatic layer plane. Therefore, it can be inferred that the defects primarily occur outside the plane in this temperature range.

The change in the parameter of peak area demonstrates that within this temperature range, the pyrolysis results in an increase in the proportion of defect structure, while the proportion of ordered graphite carbon structure decreases. This can be attributed to the poly-condensation reaction that occurs during the pyrolysis process. Eventually, the macromolecular structure of the coal will be transformed into a dense aromatic structural unit [28]. At the temperature set in the experiment, the reaction is in the primary stage. During the primary stage, the chemical bonds within macromolecular compounds are broken, resulting in the formation of small molecules. These molecules then deposit on the particle’s surface, creating numerous defective carbon structures and amorphous carbon structures. Since the sp^2^ hybridization has not yet formed, the proportion of graphitized structure is also relatively low. This result is consistent with the results obtained by Li et al. [45].

Figure 2c,d illustrate the fluctuation range (standard deviation) of coal and coke area parameters. It is observed that the standard deviation of char is generally higher than that of coal, indicating a greater dispersion in the parameters of char. The composition of coal is quite intricate. Structurally, it can be approximately represented by various models such as Fuchs, Given, Wiser, Shinn, and so on. From the perspective of macerals, coal can be categorized into vitrinite, inertinite, exinite, and other components [53]. As temperature rises, the macromolecular structure breaks down, leading to the detachment and deposition of hydroxyl, alkyl, and carbonyl groups that connect the benzene ring [45], thereby increasing the chemical structure’s heterogeneity. Moreover, at temperatures exceeding 500 °C, the interaction of macromolecular free radicals in vitrinite with alkyl and organic compounds produced during vitrinite pyrolysis generates a multi-phase intermediate product [43], further enhancing sample heterogeneity. Consequently, the study findings demonstrate that the parameter results for char exhibit higher dispersion.

**Figure 3:**
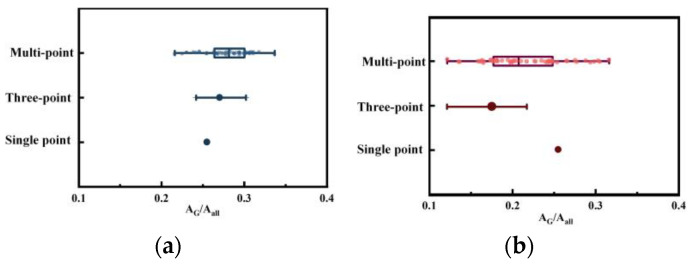
*A_G_/A_all_* distribution of single-point Raman, three-point Raman and multi-point Raman spectroscopy: (**a**) coal; (**b**) char.

Comparing the Raman parameter distribution results under different detection points can illustrate the necessity of using multi-point detection. Figure 3 also presents the distribution of micro-Raman *A_G_/A_all_* data using single-point, three-point, and multi-point methods. The single-point method alone is insufficient to represent the entire dataset. Although the three-point method expands the range of data, it fails to capture fluctuations and details. The multi-point method offers a solution to the limitations of the single-point and three-point methods. The multi-point micro-Raman technique involves scanning multiple points within a region, including both single-point and three-point scanning results. This approach considers the overall information of the sample, minimizing errors caused by the heterogeneity of coal/char in single-point scanning and potential deviations in three-point scanning. As a result, it provides a more comprehensive and accurate reflection of the diversity and complexity of the data, enabling a clearer understanding of the entire dataset. By analyzing the results of multi-point micro-Raman spectroscopy, a more accurate understanding of the changes in carbon structure during the conversion of coal to char can be achieved. This method helps to avoid the limitations of single-point and three-point methods.

### 2.2. Intensity Parameter (I)

**Figure 4:**
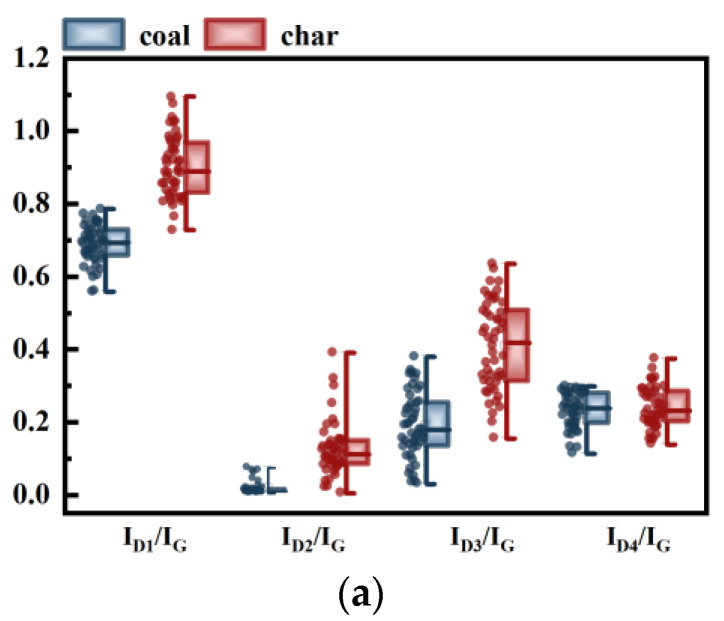

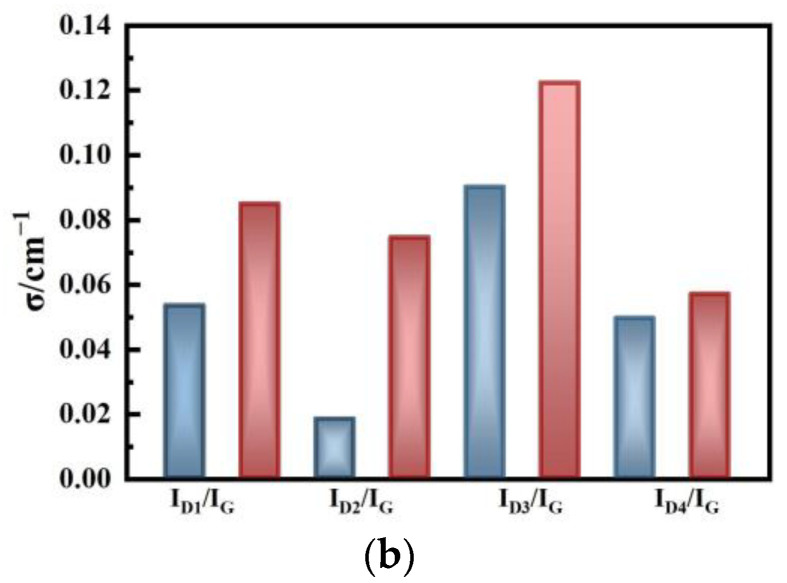
Relationship between the intensity parameters of coal and char: (**a**) changes in *I_D1_/I_G_*, *I_D2_/I_G_*, *I_D3_/I_G_*, and *I_D4_/I_G_*; (**b**) standard deviations of *I_D1_/I_G_*, *I_D2_/I_G_*, *I_D3_/I_G_*, and *I_D4_/I_G_*.

Figure 4 shows the variation relationship and standard deviation of Raman intensity parameters for coal and coke. In Figure 4a, the changes in parameters *I_D1_/I_G_*, *I_D2_/I_G_*, *I_D3_/I_G_*, and *I_D4_/I_G_* are depicted. It can be observed that, except for *I_D4_/I_G_*, the data exhibit an upward trend during the transformation of coal into char. The Raman parameters *I_D1_/I_G_* and *I_D3_/I_G_* are commonly used to represent the relative contents of defective carbon structures and amorphous carbon structures, respectively [54]. The D_2_ band appears as a shoulder on the G band, and its strength increases as the degree of graphitization increases. The increases in *I_D1_/I_G_*, *I_D2_/I_G_*, and *I_D3_/I_G_* suggest the depolymerization of the macromolecular structure and the deposition of small molecules on the carbon surface. The D_4_ peak typically represents a cross-linked structure, associated with atoms having sp^3^ hybrid orbitals. During the pyrolysis process, *I_D4_/I_G_* gradually decreases due to the depolymerization and movement of the aromatic structure, leading to fracture of the cross-linked structure between adjacent carbon crystals. Simultaneously, the cracking reaction of the aromatic group side chain dominates, resulting in the release of numerous volatile small molecules. At this temperature, the deposition exceeds the volatilization, resulting in an increase in Raman parameters towards a disordered structure, same to other findings in the literature [55,56].

**Figure 5:**
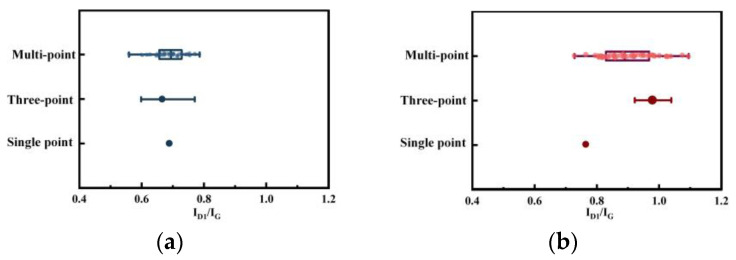
*I_D1_/I_G_* distribution of single-point Raman, three-point Raman, and multi-point Raman spectroscopy: (**a**) coal; (**b**) char.

Figure 5 presents the distribution map of single-point, three-point, and multi-point *I_D1_/I_G_* data. Analysis of the multi-point micro-Raman results includes both single-point and three-point results. Although the overall trend indicates an increase in the value of *I_D1_/I_G_* during the coal-to-char transformation process, the magnitude of this increase varies significantly. The multi-point method shows a mean value rise from 0.70 to 0.89; the three-point method shows a rise from 0.63 to 0.97; and the single-point method shows an increase from 0.69 to 0.79. These variations in data impact the extent of change in defect structure, highlighting the importance of utilizing multiple points to accurately represent the actual structural changes in the material, especially for char.

### 2.3. Full Width at Half Maximum (FWHM/W) and Position (P) Parameter

**Figure 6:**
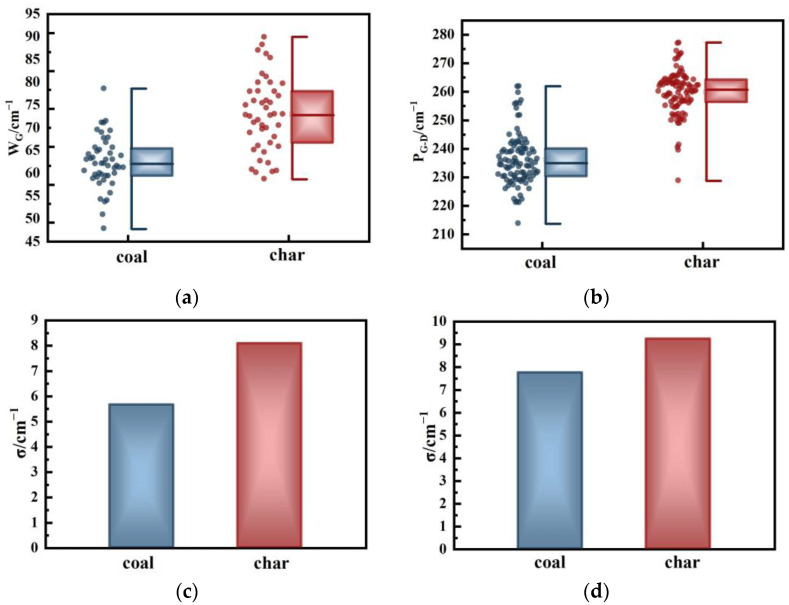
Relationship between other parameters of coal and char: (**a**) change in *W_G_*; (**b**) change in *P_G-D_*; (**c**) standard deviation of *W_G_*; and (**d**) standard deviation of *P_G-D_*.

Figure 6 presents the *FWHM* parameters and peak position difference parameters for coal and char. As coal is converted into char, both values increase. For the *FWHM*, *W_G_* is directly proportional to the H/C value [57], indicating that as *W_G_* increases, the H/C value also increases. The random distribution of these molecules leads to a decrease in the degree of graphitization. Therefore, the *W_G_* value shown in Figure 6a increases with the increase in pyrolysis, indicating an increase in the defect structure. The change in *P_D_* and *P_G_* single values alone cannot fully reflect the change in structural order [28]. Therefore, it is necessary to analyze the increased *P_G-D_* after pyrolysis. An increase in this value indicates that the shoulder between the two peaks gradually widens, indicating that pyrolysis promotes the cracking and volatilization of macromolecular compounds in coal samples within this temperature range. However, unlike the area and strength parameters, the *W_G_* and *P_G-D_* parameters did not exhibit significant expansion. This could be attributed to these two parameters reflecting changes in the overall structure of the sample rather than the detailed structure differences. The fracture and volatilization of the bridge bonds in different functional groups lead to dynamic changes in their content. However, despite this variation, the overall trend remains consistent. As a result, the two parameters of char at 700 °C do not exhibit significant widening.

**Figure 7:**
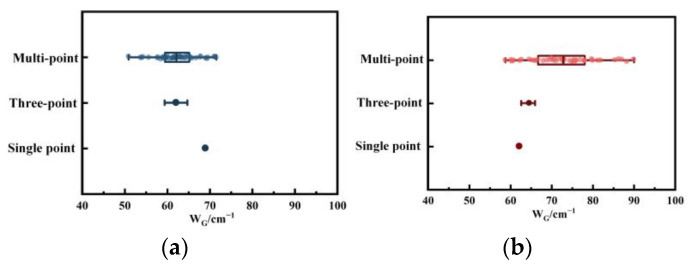
*W_G_* distribution of single-point Raman, three-point Raman, and multi-point Raman spectroscopy: (**a**) coal; (**b**) char.

**Figure 8:**
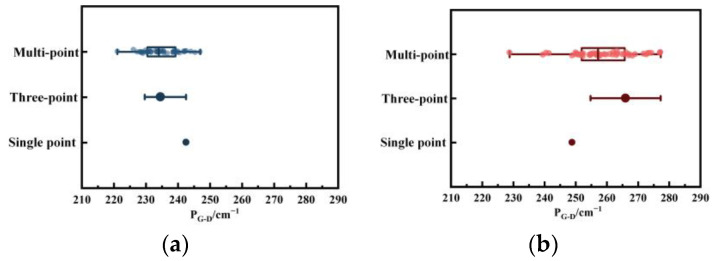
*P_G-D_* distribution of single-point Raman, three-point Raman, and multi-point Raman spectroscopy: (**a**) coal; (**b**) char.

Figure 7 and Figure 8 present the distribution characteristics of *FWHM* and peak position difference for single-point, three-point, and multi-point micro-Raman measurements. As the number of scanning points increases, the data distribution range becomes wider. It is apparent that the single-point research method fails to fully capture the structural changes in the pyrolysis process, as it only focuses on one sample point. This limitation can lead to misjudgments, where parameter changes even contrary to facts. According to Figure 7, the single point appears to show a decreasing trend in *W_G_*. However, it is important to note that the trend in this parameter is actually increasing. Additionally, although the three-point research method selects three data points for analysis, it may still not provide an accurate representation of the actual structural changes. The amplitude of the change in parameters also reflects the change in structure. In Figure 2b, the large increase in *A_D3_/A_all_* indicates that the increase in amorphous carbon structure outside the plane layer dominates in the process of disorder, which is easily overlooked by the three-point/single-point method. During the process of converting coal into char, the multi-point detection results indicate that the dataset is consistently expanding. This suggests an increased likelihood of misjudgment at single points or three points, as well as the emergence of varying trends when falling within different intervals.

When conducting single-point or three-point studies, it is crucial to carefully consider whether the chosen data points are representative and capable of fully capturing the structural changes that occur during pyrolysis. In contrast, the multi-point research method offers a more accurate reflection of the structural changes in the pyrolysis process and effectively enhances the representativeness of the data. Additionally, the selection of appropriate research methods is of utmost importance when studying the structural changes in the pyrolysis process. For detection objects such as coal char samples, it is advisable to avoid single-point research methods and instead consider three-point research methods when low accuracy requirements are acceptable. To achieve a more precise reflection of the structural changes in the pyrolysis process, the use of multi-point research methods is recommended.

### 2.4. Correlation between Different Parameters

**Figure 9:**
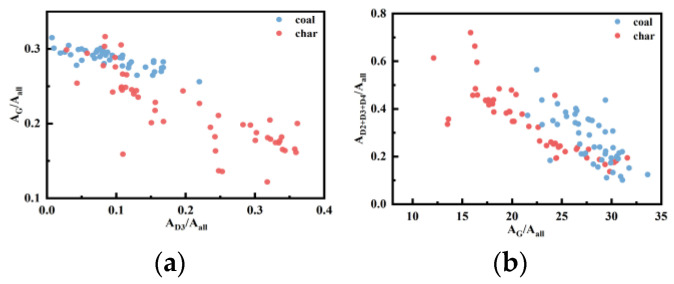

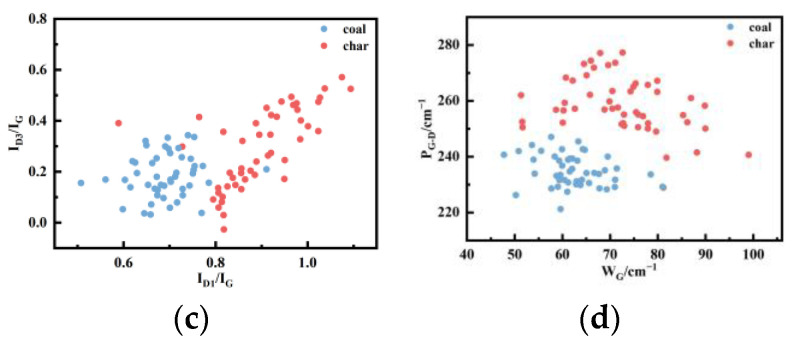
Change pattern between the parameters of different carbonaceous materials: (**a**) *A_D3_/A_all_*–*A_G_/A_all_*; (**b**) *A_G_/A_all_*–*A_D2+D3+D4_/A_all_*; (**c**) *I_D1_/I_G_*–*I_D3_/I_G_*; and (**d**) *W_G_*–*P_G-D_*.

To further explore the transformation of the structure, we compared the relationship between the parameters and selected four sets of parameters with significant changes, as illustrated in Figure 9, to provide various perspectives to analyze the carbonaceous material changes. Figure 9a depicts the correlation between *A_D3_/A_all_* and *A_G_/A_all_*. The concentration of coal data in the upper left corner of the figure suggests a significantly higher order degree of coal compared to char, with a diffusion trend from coal to char. Figure 9b demonstrates the relationship between *A_G_/A_all_* and *A_D2+D3+D4_/A_all_*. As coal converts to char, the data exhibit a linear leftward motion, indicating a gradual decrease in *A_G_/A_all_* and an increase in *A_D2+D3+D4_/A_all_*. This can be attributed to the decomposition of macromolecules into smaller molecular compounds and the rise of disordered structures during coal conversion. Figure 9c showcases the relationship between *I_D1_/I_G_* and *I_D3_/I_G_*, with the data diffusing to the upper right from coal to char, further indicating the transition of carbon structure towards disorder. Figure 9d displays the relationship between *W_G_* and *P_G-D_*. Upon conversion from coal to char, the data shift to the upper right, once again signifying the disorderliness of the material. Additionally, the overlapping level of data between coal and char is not significant, indicating that it can serve as a distinguishing factor between the two.

## 3. Mapping

**Figure 10:**
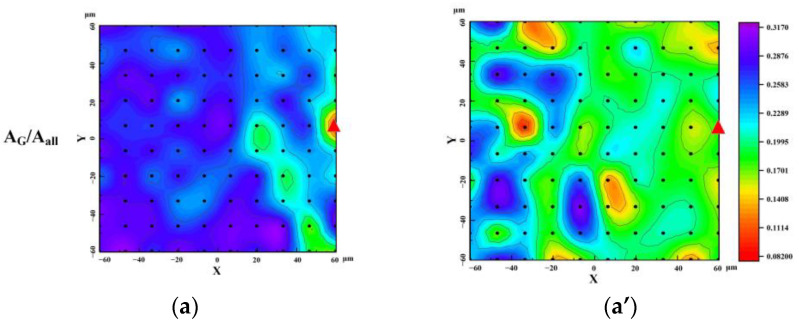

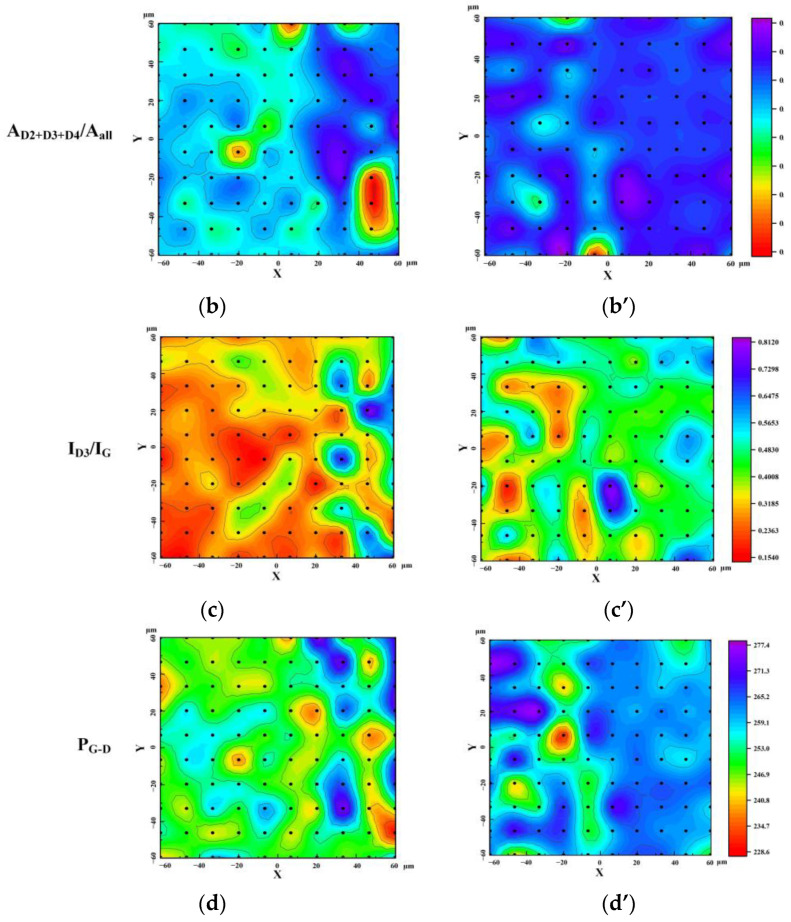
Mapping of micro-Raman parameters of coal and char during pyrolysis: (**a**) *A_G_/A_all_* of coal; (**a’**) *A_G_/A_all_* of char; (**b**) *A_D2+D3+D4_/A_all_* of coal; (**b’**) *A_D2+D3+D4_/A_all_* of char; (**c**) *I_D3_/I_G_* of coal; (**c’**) *I_D3_/I_G_* of char (**d**) *P_G-D_* of coal; and (**d’**) *P_G-D_* of char.

Figure 10 depicts the mapping imaging of Raman parameters for char and coal, illustrating that the experimental findings can, to some extent, capture the alterations in carbon structure at the micro scale before and after pyrolysis. The color intervals in the mapping diagram represent the variations in Raman parameters.

Figure 10a illustrates the Raman parameter of *A_G_/A_all_* mapping imaging in coal, where a higher value indicates a more graphitized structure. The color transition from dark blue to green in Figure 10a,a’ signifies a decrease in value, indicating a reduction in the proportion of graphitized structure. The parameter *A_D2+D3+D4_/A_all_* in Figure 10b reflects the presence of disordered carbon structure, with a color shift from red to green or blue indicating an increase in value, and thus, an increase in defective carbon structure. *I_D3_/I_G_* typically signifies the amorphous carbon structure, as seen in Figure 10c, where the red transitions to a large area of green, indicating an increase in value, and hence, an increase in amorphous carbon structure. Tromp et al. [58] describes a scheme of the free radical mechanism from coal to char. At the experimental temperature, coal is in the stage of chemical bond breaking, releasing small molecular bonds. The mapping imaging results align with those described in the section. As pyrolysis progresses, the carbon structure of coal increases the proportion of defect structure due to the breaking of chemical bonds and the formation of small molecules during the condensation of macromolecules. The results demonstrate that mapping can effectively illustrate the transformation of carbon structure during the pyrolysis process of char derived from coal. The heterogeneity of coal/char is evident from the distribution of colors. In the future, a multi-point method could be used to detect such heterogeneity materials and accurately reflect the actual results.

In addition, it is possible that the irregular deposition of fragmented small molecules, such as the triangular symbols in (a) and (a’), may explain why certain color changes deviate from the expected pattern of formation.

Admittedly, such a micron-sized area is also minimal for the entire coal sample. Our research aims to attract more and more advanced technologies to be applied to the field of coal/char with the development of technology to further solve various problems due to heterogeneity.

## 4. Materials and Methods

The experiment utilized low-rank coal with high volatile matter (37.1 wt.%) from the Boyuan coal mine in Shenmu, Shaanxi province, China. This coal has the characteristics of low degree of coalification, high reactivity, and good thermal stability. The raw coal was subjected to heating in a TL1200 tube furnace (Boyuntong Instrument Ltd., Nanjing, China) at 700 °C for 2 h, with the entire process being conducted under N_2_ protection. Subsequently, the samples were further crushed to less than 200 mesh and pressed into a wafer with a height of 3–5 mm and a diameter of 13 mm using a tablet press to address the issue of surface roughness. The proximate analysis of the samples is shown in Table 2, according to Chinese National Standard GB/T 212-2008 [59]. The obtained char has a lower volatile matter.

A LabRAM HR Evolution laser confocal Raman spectrometer (HORIBA Scientific, Kyoto, Japan) was utilized for detection, performing two-dimensional plane scanning with a Nd:YAG laser of 532 nm to excite high-quality Raman spectra of the sample. Prior to experimentation, it was verified that the Raman intensity and fluorescence intensity at this wavelength were suitable. The study focused solely on first-order Raman spectroscopy, testing a wavenumber range of 800–1800 cm^−1^. With an objective lens magnification of 10×, an acquisition area of 120 μm × 120 μm, and a step size of 10 μm, data were densely collected across the entire plane while avoiding overlap between scanning points. To balance acquisition time and sample integrity, each test point had an acquisition time of 10 s and an attenuator power of 1% was used. This study determined the optimal combination of Raman signals through testing. It was found that too short a test time may result in weak Raman signal intensity, failing to capture all sample information. Conversely, a longer test time can lead to excessive local temperatures, potentially damaging the sample surface. Inappropriate test combinations may result in abnormal changes in peak position and peak shape. Additionally, standard Raman detection procedures were conducted, including randomly single-point spectrum collection of the sample and three-point scanning spectrum collection. The parameters for test range, laser wavelength, and acquisition time remained consistent throughout. The peak of single-crystal silicon was identified at 520.7 cm^−1^ after applying silicon wafer correction data prior to detection. The detection data were then processed using the Savitzky–Golay filter in ORIGIN 2023b software to reduce high-frequency noise interference. Following this, fluorescence interference was minimized by adjusting the baseline. Lastly, normalization was carried out to eliminate the impact of laser power and aid in data integration.

## 5. Conclusions

(1) The distribution of parameters in the multi-point micro-Raman detection results provides a comprehensive demonstration of the heterogeneity of coal/char samples. As coal is converted into char, the decomposition of aliphatic side chains and aromatic structures further enhances the heterogeneity of char at 700 °C.

(2) The area parameters of Raman spectroscopy demonstrate that the graphitization of Shenmu low-rank coal is reduced due to the fracture and deposition of bridge bonds after the conversion to char. Additionally, the increase in *A_D3_/A_all_* indicates an increase in out-of-plane defects in the aromatic layer. The intensity parameters indicate that aromatic volatilization is less than deposition at 700 °C, and the decrease in *I_D4_/I_G_* suggests a fracture of the cross-linked structure at this temperature. Furthermore, FWMH provides additional insight into the disordered transformation of the carbon structure from the perspective of functional groups.

(3) The relationships between the two parameters are analyzed after coal is converted into char. By considering parameter displacement, it is observed that there is an increasing trend of disorder from coal to char. It is worth emphasizing that these conclusions may change with the change in coal type and the final temperature of char preparation.

(4) The advantage of micro-Raman surface scanning with multi-point detection is its ability to cover a large area of samples and obtain data in batches. This method effectively avoids errors caused by selecting single/three points and provides a comprehensive understanding of the overall attributes and distribution of samples. Additionally, mapping can visually display changes in structural parameters through images, allowing for a more intuitive representation of structural changes in the region. Therefore, micro-Raman multi-point detection is considered an effective and favorable method in the field of carbonaceous materials.

## Figures and Tables

**Table 1:** First-order common peak vibration modes of carbonaceous materials.

Band	Wavenumber/cm^−1^	Fitting Peak Shape	Vibration Mode
G	1580	Lorentzian	Ideal graphitic lattice (E_2g_-symmetry)
D1	1350	Lorentzian	Disordered graphitic lattice (A_1g_-symmetry)
D2	1620	Lorentzian	Disordered graphitic lattice (E_2g_-symmetry)
D3	1500	Gaussian	Amorphous carbon
D4	1200	Lorentzian	Disordered graphitic lattice (A_1g_-symmetry)

**Table 2:** Proximate analysis of the samples.

Sample	Proximate Analysis/%			
	A_ad_	M_ad_	FC_ad_	V_ad_
Coal	8.6	3.8	52.2	37.1
Char	6.3	1.3	79.1	8.7

## Data Availability

The original contributions presented in the study are included in the article and Supplementary Material, further inquiries can be directed to the corresponding author.

## Supplementary Materials

The following supporting information can be downloaded at: https://www.mdpi.com/article/10.3390/molecules29102361/s1, Figure S1. The FTIR spectra of coal and char. References [60,61,62,63,64] are cited in the supplementary materials.
