# Supplementary material for: Analysis of Structural Heterogeneity in Low-Rank Coal and Its Pyrolyzed Char Using Multi-Point Scanning Micro-Raman Spectroscopy
Source: Molecules. 2024 May 17;29(10):2361. doi: 10.3390/molecules29102361 (PMC11124247; doi:10.3390/molecules29102361)
Supplement: Supplementary file 1 [file molecules-29-02361-s001.zip › molecules-2988322-supplementary.pdf]

Supplementary Materials: A Fourier transform infrared detection method was employed for auxiliary detection (Thermo Scientific Nicolet iS20). Prior to the experiment, the dried coal sample was combined with potassium bromide at a ratio of 1:100. The instrument's scanning time was set to 32 seconds. The acquisition wavenumber was selected within the range of 400-4000 cm^{-1} [1-3]. Figure S1 illustrates the changes in characteristic peaks of coal and char. The intensity of the characteristic peaks in the 2800-3000cm^{-1} interval (aliphatic C-H stretching region) and the 1500-2800cm^{-1} interval (C = O, aromatic C = C stretching region) decreased significantly, indicating the removal of side chains and heterocycles in coal during pyrolysis. Additionally, a noticeable reduction in functional group types is observed in the 400-1300cm^{-1} interval (fingerprint region), suggesting that pyrolysis at 700 °C promotes the fracture and decomposition of chemical bonds [4]. The peak of -OH in the range of > 3040 cm^{-1} does not show a significant decrease with increasing temperature. This could be due to the absorption of moisture by the char during grinding with KBr powder [5].

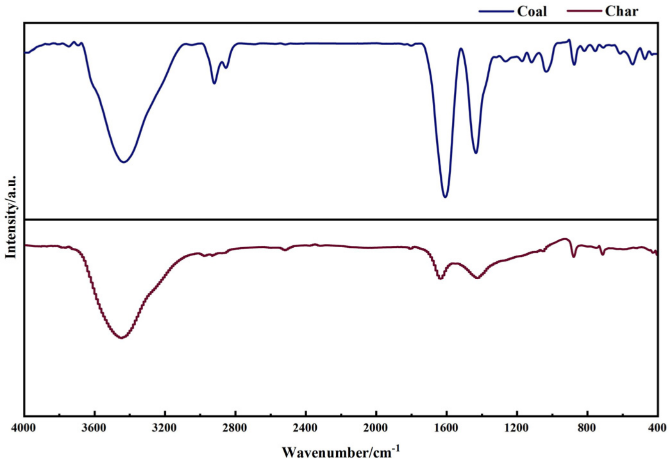

Figure S1. the FTIR spectra of coal and char.

1. Van, H.K.H. Progress of coal science in the 20th century. *Fuel* **2000**, *79*, 1–26.
2. Li, W.; Zhu, Y.M. Structural characteristics of coal vitrinite during pyrolysis. *Energy Fuels* **2014**, *28*, 3645–3654.
3. Sonibare, O.O.; Haeger, T.; Foley, S.F. Structural characterization of nigerian coals by X-ray diffraction, Raman and FTIR spectroscopy. *Energy* **2010**, *35*, 5347–5353.
4. Niu, Z.Y.; Liu, G.J.; Yin, H.; Wu, Dun,; Zhou, C.C. Investigation of mechanism and kinetics of non-isothermal low temperature pyrolysis of perhydrous bituminous coal by in-situ FTIR. *Fuel* **2016**, *172*, 1–10. https://doi.org/10.1016/j.fuel.2016.01.007.
5. Chen, C.; Gao, J.S.; Yan, Y.J. Observation of the type of Hydrogen Bonds in Coal by FTIR. *Energy Fuels* **1998**, *12*, 446–449.
